# Temporal dissociation of phencyclidine: Induced locomotor and social alterations in rats using an automated homecage monitoring system – implications for the 3Rs and preclinical drug discovery

**DOI:** 10.1177/0269881120920455

**Published:** 2020-05-21

**Authors:** Emma J Mitchell, Ros R Brett, J Douglas Armstrong, Rowland R Sillito, Judith A Pratt

**Affiliations:** 1 Strathclyde Institute of Pharmacy and Biomedical Sciences, University of Strathclyde, Glasgow, UK; 2 School of Informatics, University of Edinburgh, Edinburgh, UK; 3 Actual Analytics Ltd, Edinburgh, UK

**Keywords:** Homecage, welfare, locomotor activity, social interactions, refinement, phencyclidine, schizophrenia, autism

## Abstract

**Background:**

Rodent behavioural assays are widely used to delineate the mechanisms of psychiatric disorders and predict the efficacy of drug candidates. Conventional behavioural paradigms are restricted to short time windows and involve transferring animals from the homecage to unfamiliar apparatus which induces stress. Additionally, factors including environmental perturbations, handling and the presence of an experimenter can impact behaviour and confound data interpretation. To improve welfare and reproducibility these issues must be resolved. Automated homecage monitoring offers a more ethologically relevant approach with reduced experimenter bias.

**Aim:**

To evaluate the effectiveness of an automated homecage system at detecting locomotor and social alterations induced by phencyclidine (PCP) in group-housed rats. PCP is an N-methyl-D-aspartate (NMDA) receptor antagonist commonly utilised to model aspects of schizophrenia.

**Methods:**

Rats housed in groups of three were implanted with radio frequency identification (RFID) tags. Each homecage was placed over a RFID reader baseplate for the automated monitoring of the social and locomotor activity of each individual rat. For all rats, we acquired homecage data for 24 h following administration of both saline and PCP (2.5 mg/kg).

**Results:**

PCP resulted in significantly increased distance travelled from 15 to 60 min post injection. Furthermore, PCP significantly enhanced time spent isolated from cage mates and this asociality occured from 60 to 105 min post treatment.

**Conclusions:**

Unlike conventional assays, in-cage monitoring captures the temporal duration of drug effects on multiple behaviours in the same group of animals. This approach could benefit psychiatric preclinical drug discovery through improved welfare and increased between-laboratory replicability.

## Introduction

Rodent behavioural assays are used extensively to gain insights into disease mechanisms and predict the efficacy of putative treatments. A wealth of paradigms are available to assess a broad range of behaviours including sensorimotor function, sociability, depression, anxiety and cognition (Crawley, 2007; Wahlsten, 2011). However, conventional behavioural assays have several limitations that may impact welfare and replicability (Kafkafi et al., 2018). Firstly, testing often involves separating the animal from its cage mates and placing it in an unfamiliar apparatus; however disrupting a rodents social environment causes stress-associated behavioural, hormonal and neurochemical changes that could confound data interpretation (Beery and Kaufer, 2015; Ferland and Schrader, 2011; Ieraci et al., 2016). Furthermore, for the same behavioural task, phenotypic outcome can vary depending on the laboratory environment, experimenter identity and handling/habituation techniques utilised (Chesler et al., 2002; Gouveia and Hurst, 2013; Sorge et al., 2014; van Driel and Talling, 2005; Wahlsten et al., 2003). For convenience, behavioural assessments are often conducted during the light period which represents the natural resting phase for rodents; however, animals are less susceptible to stress when tested in the dark (Aslani et al., 2014; Hawkins and Golledge, 2018; Kelliher et al., 2000). Many assays focus on a discrete behavioural domain; however when examining drug effects, multiple interacting behaviours with distinct temporal profiles can occur leading to potentially skewed conclusions and an incomplete understanding of the profile of action. To improve animal welfare, obtain more reliabile reproducible results and gain richer data sets, it is important to resolve the above issues (Kafkafi et al., 2018).

Recent emphasis has been placed on the development of automated homecage systems for more ethologically relevant testing and reduced experimenter bias (Richardson, 2012, 2015). Unlike conventional assays, automated approaches permit continual monitoring for days to weeks, even during the dark phase and they reduce the requirement for handling which is a source of stress and outcome variation (Gouveia and Hurst, 2013; Hurst and West, 2010). Several homecage technologies are available for the automated tracking of locomotor activity, circadian rhythmicity, social interactions and feeding behaviour (Clemmensen et al., 2015; de Visser et al., 2006; Freund et al., 2013; Galsworthy et al., 2005; Morretti et al., 2005; Robinson and Riedel, 2014; Vannoni et al., 2014). However, many of the existing systems involve bespoke semi-natural environments and they have limited scope for acquiring the social activity of more than two animals (see Bains et al., 2018 for a review). Objective assays for monitoring social behaviours are crucial for screening therapeutic candidates for a range of neuropsychiatric disorders including schizophrenia and autism (Mier and Kirsch, 2017; Worley and Matson, 2012). Traditionally, sociability is assessed by hand-scoring encounters between pairs of rodents in an arena (or homecage): complex interactions (e.g. allogrooming, pouncing, wrestling) can be measured but manual scoring is laborious, often limited to a short (~10 min) window and subject to experimenter bias (Boulay et al., 2004; Cox & Rissman, 2011; Himmler et al., 2013; Lee et al., 2005; Paasonen et al., 2017; Sato et al., 2013). Another technique involves utilising a three-chambered arena and measuring the length of time a rodent spends in the chamber containing a caged conspecific; this task can be automated for an objective high-throughput approach but testing is often restricted to a 5–10 min trial performed under bright lighting (Hanks et al., 2013; McKibben et al., 2014; Moy et al., 2013; Nadler et al., 2004).

ActualHCA^TM^ (Actual Analytics, UK) was developed as part of the ‘Rodent Big Brother’ project funded by the National Centre for the Replacement, Reduction and Refinement of Animals in Research, UK (NC3Rs) to continually monitor multiple rodents within the homecage in the absence of invasive surgery (Bains et al., 2016). This technology is compatible with conventional individually ventilated caging (IVC) and capable of collecting spatial data for each individual animal within the cage 24 h per day without experimenter intervention. ActualHCA^TM^ permits individual locomotor, social and temperature data to be acquired from group-housed animals across days to weeks (Bains et al., 2016, 2018; Redfern et al., 2017). In rats, ActualHCA^TM^ can accurately detect alterations in locomotion and temperature induced by sedative and stimulant agents (chlorpromazine, clonidine and amphetamine) (Tse et al., 2018). However, it is yet to be established whether ActualHCA^TM^ can detect social changes induced by a pharmacological agent.

Here, in groups of Lister-Hooded rats, we explored the effectiveness of ActualHCA^TM^ at detecting behavioural deficits associated with phencyclidine (PCP). N-methyl-D-aspartate (NMDA) receptor hypofunction is implicated in the pathogenesis of schizophrenia and accordingly, NMDA receptor antagonists including PCP are widely used to model aspects of schizophrenia in animals (Jones et al., 2011; Morris et al., 2005; Pratt et al., 2012, 2018). Our main objective was to validate the ability of ActualHCA^TM^ to accurately detect the locomotor and social effects of PCP and establish the temporal profiles of these behaviours. In addition, we investigated the possibility that PCP impacts circadian activity. We propose that utilisation of the ActualHCA^TM^ system in concurrence with conventional assays may facilitate preclinical drug discovery via improved animal welfare, increased data reproducibility and the provision of richer data sets that permit simultaneous assessment of a range of behaviours.

## Materials and methods

### Ethical statement

All procedures were approved by the Animal Welfare and Ethical Review Body of the University of Strathclyde and were conducted in accordance with the Animal Scientific Procedures Act, 1986 (Project Licence PPL 60/4463). The ARRIVE guidelines were utilised in the execution and reporting of this experiment (Kilkenny et al., 2010).

### Animals, housing and husbandry

Fifteen male Lister-Hooded rats (Charles River Laboratories, Margate, UK; aged 40–58 days during data acquisition) were housed in groups of three in IVC cages (Tecniplast, Buguggiate, Italy) for 1 week before experimentation commenced. Cages contained a 1–1.5 cm layer of Aspen chip bedding along with sizzle nesting and chew bricks (Datesand, UK) for environmental enrichment. Rats were maintained under a 12 h light/dark cycle (lights on at 06:00) with food and water available *ad libitum*. Monitoring of health and well-being was performed daily during husbandry procedures.

### Microchip implantation

Rats were briefly anaesthetised with isoflurane in 100% oxygen (Baxter Healthcare Ltd, UK: 4% for chamber induction; 1–2% for facemask maintenance). The metacarpal region of the hind-foot was pinched to confirm appropriate depth of anaesthesia then a radio frequency identification (RFID; BIO13.THERM.03V1; Biomark Inc., USA) tag was inserted subcutaneously into the lower right abdominal quadrant using a sterile implant device (a modified syringe supplied by the RFID manufacturer). Rats were then returned to their homecages and allowed to recover from the microchipping procedure for 3 days prior to data acquisition. There were no welfare issues associated with the implantation.

### The ActualHCA^TM^ system

The ActualHCA^TM^ system is described in detail elsewhere (Redfern et al., 2017). Briefly, an IVC cage was positioned on top of an RFID reader baseplate (with 12 antennae in a 3 × 4 array) for automated acquisition of the spatial location of each individual rat. A side-mounted camera allowed for continuous video capture (day and night). Health status checks were performed daily (9am). The software package ActualHCA-Capture (Actual Analytics Ltd, UK) collected baseplate data to provide the following measures for each individual animal (in 5 and 15 min segments): distance travelled (mm) and time spent isolated (>200 mm) from all other cage mates(s). A value of > 200 mm was selected to reflect isolation time according to previous studies using conventional social interaction tests (Sams-Dodd 1996, 1998). A second software package, ActualHCA Analysis tool v 2.2.2 (Actual Analytics Ltd, UK), was utilised to overlay videos with baseplate rat location data such that individual rats could be identified during manual behavioural scoring.

### Drug administration and data acquisition

Drug administration was performed according to Procedures With Care guidelines (procedureswithcare.org.uk). For each rat, we collected ActualHCA^TM^ data for 24 h following both saline (0.9% NaCl) and PCP (2.5 mg/kg dissolved in 0.9% NaCl) administration (within subjects design). Each rat within a homecage of three animals received the same treatment regime whereby saline was administered followed by PCP 48 h later. We selected a PCP dose of 2.5 mg/kg based on our previous data and reports of deficits in conventional locomotor and social assays (Adams et al., 2013; Egerton et al., 2005; Paasonen et al., 2017; Sturgeon et al., 1979). Injections were performed 15 min prior to the onset of the dark period (17:45; intraperitoneal (IP), 4 mL/kg injection volume) in order to capture drug effects when rats are typically most active. To verify PCP– induced social deficits, conventional manual scoring of interactions (pouncing, pinning, allo-grooming, boxing and wrestling) was performed using ANVIL software (v5.1.9, Kipp) on video footage acquired 0–60 min following saline/PCP administration. The experimenter was blinded to treatment groups during manual scoring.

### Statistical analysis

Drug effects on automated ActualHCA^TM^ data were explored via repeated measures ANOVA with treatment, cage and time as factors (with each individual subject nested within its cage number) followed by Fisher’s post-hoc testing (Minitab v.18.1, State Collegue, PA, USA). Drug effects on manual social observations were explored via paired *t*-tests.

## Results

### Effect of PCP on locomotor activity

**Figure 1.**
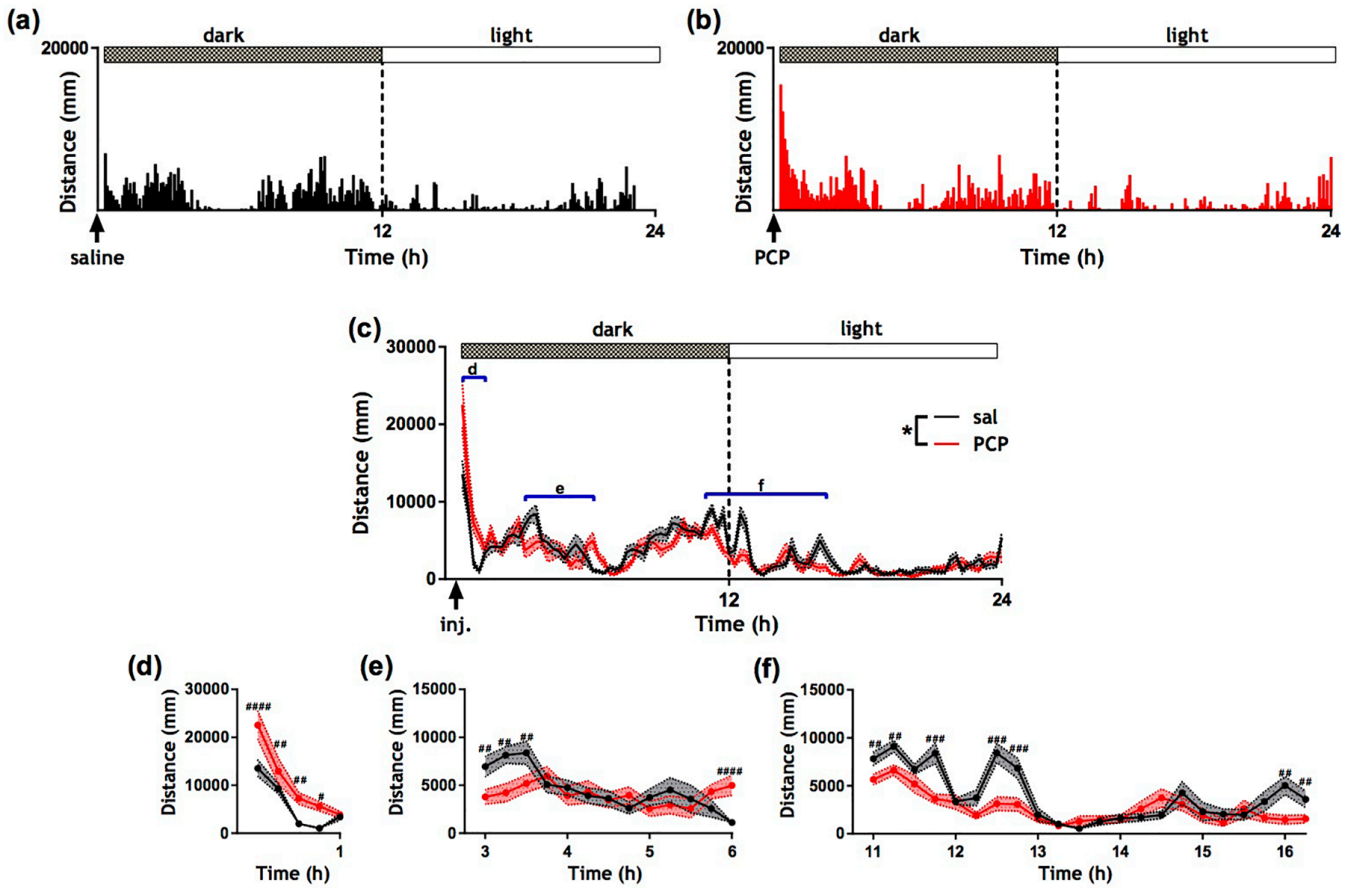
Effect of acute phencyclidine (PCP) administration on automatically-derived locomotor activity. Activity data from a randomly selected rat presented as raster plots of the sum of distance travelled (mm) in 5 min time bins over 24 h following (a) saline and (b) PCP administration. (c) Mean distance travelled presented in 15 min bins across 24 h post-treatment. Dotted lines represent the SEM. Graphs (d), (e) and (f) correspond to the regions highlighted by blue bars in (c). * *p* < 0.05 (ANOVA); ^#^*p* < 0.05; ^##^*p* < 0.01; ^###^ *p* < 0.001; ^####^*p* < 0.0001 (post-hoc Fisher test). *N* = 15 per group.

Figure 1(a) and (b) show distance travelled per 5 min bin across 24 h following saline and PCP treatment, respectively, for a single randomly selected rat. A striking burst of hyperactivity was observed immediately following PCP administration and this phenotype lasted for approximately 1 h. Apart from the initial burst of hyperlocomotion, the activity profile of PCP was remarkably similar to that of saline. Figure 1(c) shows distance travelled per 15 min bin across 24 h following saline (and PCP) treatment for all rats (mean ± SEM). There were significant main effects of treatment (*F* (1, 2870) = 6.49; *p* < 0.05), dark/light period (*F* (1, 2870) = 811.67; *p* < 0.0001) and time (*F* (94, 2870) = 16.47; *p* < 0.0001) and a treatment by time interaction (*F* (94, 2870) = 3.84; *p* < 0.0001). According to Fisher’s post-hoc analysis, PCP treated rats were hyperactive from 15 to 60 min post injection (*p* < 0.05) (Figure 1(d)). We also found evidence of hypoactivity in PCP-treated rats at 3 h post injection (*p* < 0.01) (Figure 1(e)) which may represent a recovery period following locomotor stimulation. PCP treated rats exhibited a brief bout of hyperactivity at 6 h post treatment (*p* < 0.0001) (Figure 1(e)). Additionally, PCP-treated rats showed significantly reduced activity 1 h prior to the light period (*p* < 0.01) and 45–60 min after the light period onset (*p* < 0.001) (Figure 1(f)) which could reflect a subtle drug effect on circadian rhythmicity. Finally there was a short bout of hypoactivity in PCP-treated rats at 16 h post administration (*p* < 0.01).

In summary, there was a marked short-lived (~1 h) period of PCP-induced hyperactivity followed by periods of hypoactivity, particularly just prior to and following the onset of the light period (~11 h post drug administration).

### Effect of PCP on social activity

**Figure 2.**
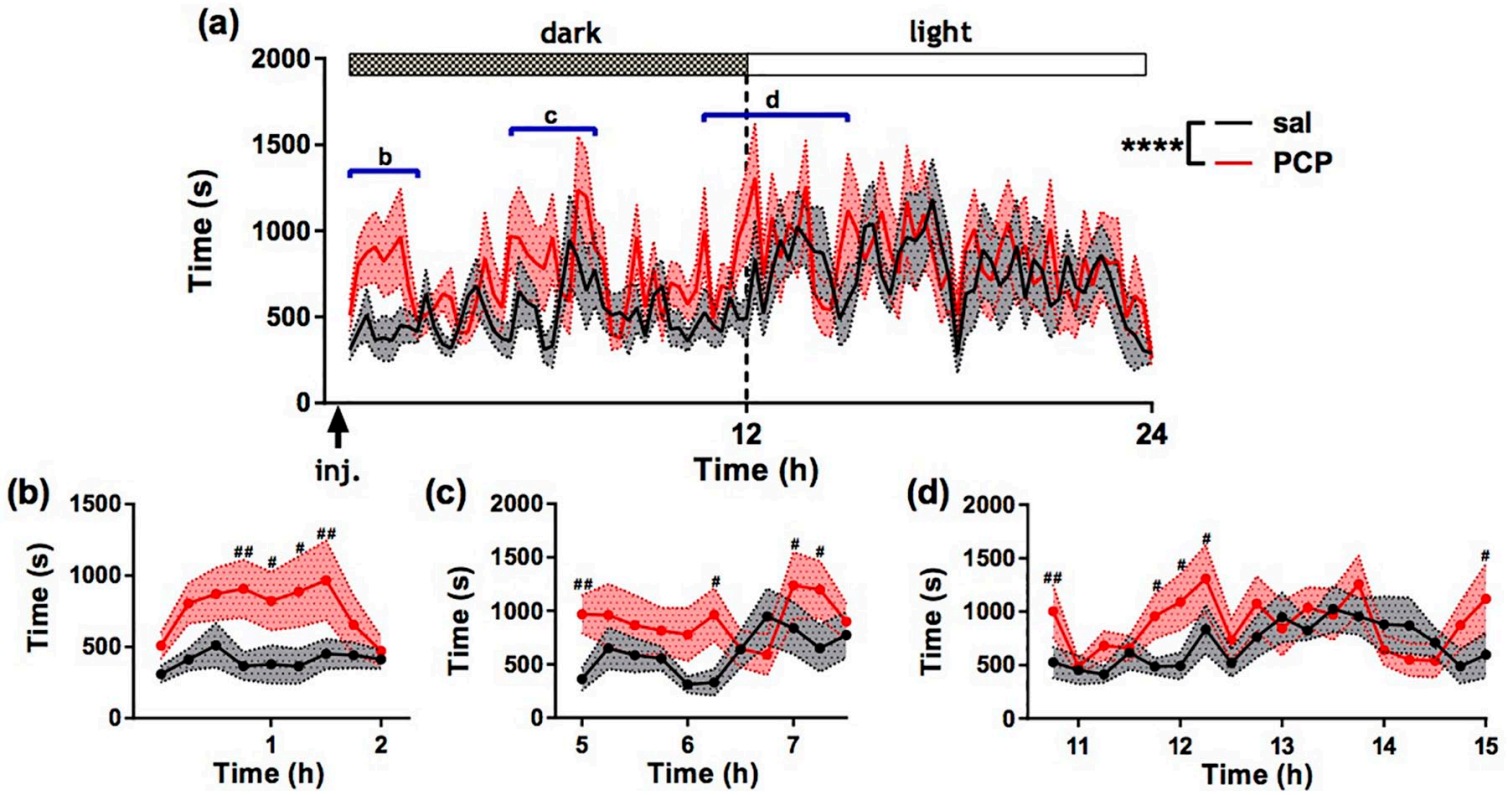
Effect of acute phencyclidine PCP administration on automatically derived social activity. (a) Time spent isolated (> 200 mm) from all cage mates(s) presented in 15 min bins across 24 h post treatment. Dotted lines represent the SEM. Graphs (b), (c) and (d) correspond to the regions highlighted by blue bars in (a). ****p < 0.0001 (ANOVA); ^#^*p* < 0.05; ^##^*p* < 0.01 (post-hoc Fisher test). *N* = 15 per group. Data represent mean ± SEM.

Figure 2 (a) shows time spent isolated (>200 mm) from all cage mates(s) per 15 min bin over 24 h following treatment for all rats (mean ± SEM). There were significant main effects of treatment (*F* (1, 2870) = 30.33; *p* < 0.0001), dark/light period (*F* (1, 2870) = 32.37; *p* < 0.0001) and time (*F* (94, 2870) =1.38; *p* < 0.01) and a treatment by time interaction (*F* (94, 2870) = 2.06; *p* < 0.0001). PCP-treated rats spent significantly more time in isolation compared to saline-treated rats from 60 to 105 min post injection (*p* < 0.05, Fisher’s post-hoc) (Figure 2(b)). We also obtained evidence of significantly increased isolation in PCP-treated rats from 5 to 7.5 h post treatment (Figure 2(c)) and during the light/dark transition phase (p < 0.05) (Figure 2 (d)).

Using conventional manual scoring, we verified PCP-induced asociality, whereby rats exhibited significant reductions in time spent allogrooming, pouncing and pinning compared to those treated with saline (Table 1) during the first hour post injection.

**Table 1.** Effect of phencyclidine (PCP) on manually scored social interactions occurring 0–60  min post injection.

Behaviour	Treatment	
	Saline	PCP
Allogrooming	24.36 ± 5.87	4.68 ± 4.59****
Anogenital sniffing	3.01 ± 1.14	0.66 ± 0.27
Pinning	13.83 ± 3.85	0.42 ± 0.28***
Pouncing	4.05 ± 0.75	0.43 ± 0.28**
**Total**	**45.31 ± 6.70**	6.19 ± 4.72****

** *p* < 0.01; ***p < 0.001; ****p < 0.0001 (paired *t*-test). *N* = 15  per group. Data represent mean ± SEM for time(s) engaged in behaviour.

## Discussion

Automated homecage analysis allows multiple behaviours to be acquired simultaneously thereby minimising the confounding influences of environmental perturbations or the presence of an experimenter. In the current study, ActualHCA^TM^ detected both hyperactivity and social isolation in rats following an acute injection of PCP; such deficits are consistent with the known pharmacological profile of PCP in rats. Thus, our findings support the use of ActualHCA^TM^ as a sensitive screening approach for pharmacological agents. This technology could contribute to improvements in drug discovery through increased reproducibility of data, detection of the concurrent temporal profile of behaviours as well as reduced and refined animal use.

### PCP-induced hyperactivity

Traditionally, drug effects on locomotion are examined by placing a single animal in an open-field (or bespoke cage) for a limited duration and quantifying its movements either automatically (e.g. video tracking or infrared beams) or manually (e.g. hand-scoring of line crossings). Using such assays, acute administration of a low dose of PCP has been shown to result in hyperactivity in rodents and this phenotype is frequently used to model positive symptoms of schizophrenia (Balu, 2016; Pratt et al., 2012, 2018). Here, automated analysis allowed us to evaluate the locomotor effects of PCP over 24 h and, apart from brief restraint for drug administration, rats remained undisturbed in their familiar homecage and social groups. PCP administration at 2.5 mg/kg induced a rapid increase in locomotor activity that reached maximum level 15 min post treatment and persisted for 60 min. This finding is consistent with results from open-field studies, showing that a dose of 2.5 mg/kg produces peak hyperactivity between 15 and 60 min (Adams et al., 2013) and between 5 and 75 min (Sturgeon et al., 1979). Unlike conventional out-of-cage locomotor assays, homecage analysis permitted a 24 h profile of PCP. At 3 h post PCP injection, rats were hypoactive for 45 min which may reflect a recovery period following locomotor stimulation. PCP-treated rats were also hypoactive during the dark/light transition phase, suggestive of altered circadian activity. The significance of these longer term reductions in locomotor activity is unclear, but a possible PCP-induced disruption of circadian rhythms warrants further investigation.

Other homecage systems utilised for the automated acquisition of rodent locomotor activity include PhenoRack (Aniszewska et al., 2014), LABORAS (Goulding et al., 2008), Phenotyper (de Visser et al., 2006; Loos et al., 2014), PhenoMaster (Clemmensen et al., 2015; Edelsbrunner et al., 2009) and Intellicage (Galsworthy et al., 2005; Krackow et al., 2010). The Phenotyper and Intellicage are effective at detecting locomotor inhibitory effects of apomorphine, and the Phenotyper can detect decreased activity associated with a high dose of PCP (Robinson and Riedel, 2014). However, apart from Intellicage, existing automated systems require animals to be solitary housed. Intellicage permits up to 16 animals per cage, deriving locomotor output from frequency of visits to water-bottle corners (as opposed to absolute distance travelled) once animals have been habituated to its bespoke environment. In contrast, ActualHCA^TM^ offers the ability to discriminate distance travelled for individual animals housed in groups in the homecage itself. In summary,when compared to other systems, the ActualHCA^TM^ offers an advantage in that drug-induced behaviours in group-housed animals in a homecage can be monitored. For the first time we have shown the 24 h profile of low dose PCP-induced locomotor hyperactivity in an automated system where animals are not solitary housed.

### PCP-induced social isolation

Rodent social paradigms typically involve placing unfamiliar pairs of rodents in a novel environment (e.g. an open field or three-chambered arena) and capturing their interactions for a brief time window either manually or with tracking software. In rats, a low dose of PCP reduces social interactions (e.g. sniffing, allogrooming) and time spent in proximity to a caged conspecific and these deficits are often claimed to be relevant to negative symptoms of schizophrenia (Gururajan et al., 2010; Sams-Dodd, 1996; Wilson and Koenig, 2014) but arguably are also relevant to social cognition deficits (Millan and Bales, 2013). In-cage monitoring allowed us to examine the social effects of PCP across an extended period of time without separating rats from their cage mates or removing them from their homecage. For the first time, using this technology, we observed a disruption of social interaction at 60–105 min post administration of 2.5 mg/kg PCP. We also found bouts of increased isolation throughout the dark period and early light period. Previous studies which measure a ‘snapshot’ of behaviour (~10 min) demonstrated reduced pairwise interactions between rats in an arena 45 min after 1.5 mg/kg (Paasonen et al., 2017) and 30 min after 1 mg/kg (Boulay et al., 2004) PCP administration. Notably at these early time points, hyper locomotor activity may be a potential confound in the interpretation of the social data. Intriguingly, the minimum dose of PCP required to disrupt social approach in the three-chamber task was reported to be 5 mg/kg (McKibben et al., 2014). Therefore the spontaneous interactions observed in the homecage may offer a greater sensitivity than some conventional assays to detect subtler social effects associated with low drug doses. Nevertheless, further optimisation of the automated home cage monitoring system is important since at present, algorithms that enable distinct elements of social behaviour (e.g. allogrooming and pinning) to be determined automatically are in the early stages of development.

We observed PCP-induced deficits in social behaviours using both automated measures of social isolation and manually scored measures of sociability. An advantage of automated measures is that it permits behavioural analysis for longer time periods (days rather than minutes) and reduces the labour-intensive process of manual recordings which are also subject to experimenter bias. Furthermore, automated recordings, are made in group-housed animals in a home cage, providing a more ethological based-analysis of social behaviours than the classic social interaction and three-chamber test where animals are separated from their cage mates.

A key finding of the present data was the distinct temporal trajectories of PCP-induced changes in hyperactivity and social deficits, but which overlapped at 60 min post-PCP treatment. Importantly, the social isolation induced by PCP outlasted the hyperactive phenotype by ~45 min, supporting the concept that the social deficits were not confounded by the locomotor hyperactivity measure. Thus, homecage analysis offers the opportunity to directly compare the onset and duration of drug effects on different behavioural domains simultaneously in the same group of animals.

It should be noted that other homecage systems exist with the capacity to monitor social activity. For example, Shemesh and colleagues (2013) developed a technology capable of discriminating interactions between three or more fluorescently labelled mice over several consecutive dark phases.

### Implications for 3Rs and drug discovery

Traditional behavioural paradigms typically require large numbers of rodents to compensate for variations in behaviour associated with environmental perturbations and handling. With the approach described here, data is collected within the homecage environment with minimal disturbance thereby potentially increasing between-laboratory reproducibility and reducing the number of animals required. While using a homecage monitoring approach satisfies the ‘reduction’ element of the 3Rs, some sources of variability (e.g. inter-animal differences) are unavoidable and therefore, a certain number of animals will always be required to ensure studies are sufficiently powered.

Furthermore, a range of behaviours can be monitored simultaneously therefore reducing the requirement of animals to undergo multiple test batteries, thereby addressing the ‘refinement’ element of the 3Rs. From a drug-discovery perspective, there is an opportunity to reveal the onset and duration of differential drug effects on distinct behaviours in the same animals simultaneously. As well as providing a richer data set, this also enables possible confounds of one behavioural effect upon another to be revealed. Additionally, since a large quantity of longitudinal data is acquired which increases statistical power, fewer rodents may be required to detect drug effects.
